# A Brief History of the Medical Milk Commission in the United States

**Published:** 1912-06

**Authors:** C. A. Rhodes

**Affiliations:** Atlanta, Ga.


### A BRIEF HISTORY OF THE MEDICAL MILK COMMISSION IN THE UNITED STATES.

By C. A. Rhodes, M. D., Atlanta, Ga.

The birth of the Medical Milk Commission dates back some twenty-two years, when in 1890 the New Jersey Medical Society, which is the oldest medical organization in the United States, under the leadership of Dr. Henry L. Coit, appointed a committee of forty physicians to make an investigation of the milk supply as far as it affected the public health. The committee's report showed the direct relation between mortalities and milk, and for two years tried to get legislation for a strict scientific supervision of all the dairies in the State, and thus improve the character of the milk. The effort resulted in little except to arouse the profession to the importance of the subject of pure milk, and through the press to stimulate the laity to a widespread interest in the dangerous character of impure milk, and to call to the attention of the philanthropists the opportunity to devote their money to the saving of infants' lives through the Milk Depots or Milk Dispensaries.

Having failed to obtain prompt relief through the law, which, as a rule, operates slowly, another plan was adopted whereby the physicians themselves might supervise the production of milk and thus be perfectly sure of its purity. This plan was submitted to and endorsed by the Practitioners' Club, in Newark, in December, 1892, and a search was begun for a dairy with suitable equipment to carry out such rigid regulations. After a six months' search one was found—that of Mr. Stephen Francisco, of Caldwell, New Jersey, who was the first to sign so stringent a contract, and to assume the obligation to conform to the dictation of a Medical Milk Commission, and on April 13, 1893, the production of what is known as "Certified Milk" was begun.

The purpose of this Commission was stated as follows:

"The objects of the Commission are to establish correct clinical standards of purity for cow's milk; to become responsible for a periodic inspection of the dairies under its patronage; provide for chemical and bacteriological examinations of the product, and the frequent scrutiny of the stock by a competent veterinarian; to promote only professional and public interests."

The following are three general requirements or standards for the milk: (1), an absence of large numbers of micro-organisms, and the entire freedom of the milk from pathogenic varieties; (2), unvarying resistance to early fermentative changes in the milk, so that it may be kept under ordinary conditions without extraordinary care. (3), a constant nutritive value of known chemical composition, and a uniform relation between the percentages of fat, proteid, and carbohydraets."

To follow the above requirements three principal rules are necessary:

"First: that physicians give their practical support to an effort conducted by a medical commission selected by a medical society which shall endeavor to bring to the city a supply of milk produced under such regulations that purity shall be assured.

Second: that approved and trustworthy dairymen possessing honor, financial ability, and dairy facilities, shall be induced by promise of medical support and the increased price of their milk to conduct their dairies, collect and handle the milk in conformity with the contract required by the Medical Commission and imposed by it in due legal form.

Third: that the duties of the Commission shall be: first, to establish correct clinical standards of purity for cow's milk; second, to be responsible for a periodical and personal inspection of the dairy or dairies under its patronage; third, to provide for frequent expert examination of the stock by competent and approved veterinarians, and for medical supervision of the employees by competent physicians. The milk produced shall also be subjected to periodical chemical analysis, and to bacterial counts under the direction of the Commission as often as in its judgment is desirable.

The experts employed by the Commission shall render their reports to this body, which constitute the basis of its certification of the product. The expense of examinations and inspections shall be defrayed by the dairymen, but the members of the Commission shall receive no pay for their services.

The findings of the Commission shall be published to the profession only, and the milk thus produced shall be known as "Certified Milk," and be sold in quart containers bearing the date of milking and the seal of the Commission.

The first commission, as previously stated, was organized April 13, 1893; five years later (1898), the second was formed. The movement spread more rapidly after 1898, as the following statistics will show:

In 1900, one; 1901, one; 1902, five; 1903, two; 1904, three; 1905, one; 1906, five; 1907, six; 1908, eleven; 1909, five; 1910, four, and as yet we have no report of 1911, but it is safe to say in all about eighty commissions are organized or in the process of organization.

In February, 1907, the Cincinnati Commission, of which Dr. Otto P. Geier was Secretary, suggested that the various Milk Commissions hold a conference in connection with the meeting of the American Medical Association at Atlantic City. A temporary organization was formed. Dr. Henry L. Coit, Dr. Otto P. Geir, Dr. Samuel Mc D. Hamill, Dr. Rowland G. Freeman, Dr. William H. Park, and Dr. Thomas W. Harvey, acting as a Committee, formulated a program and called the conference for June 3rd, 1907, at Atlantic City.

This Conference was remarkable, as delegates were present from twelve different States, representing twenty-one commissions in as many cities. Over 100 leading hygienists and physicians attended this meeting, much work was accomplished, which resulted in a permanent organization to be known as the American Association of Medical Milk Commissions. The second annual meeting was held in Chicago, June 1st, 1908. The third at Washington, D. C.; the fourth at St. Louis, June 6th, 1910. Up to this time these meetings were held on the day previous to the meeting of the American Medical Association. However, the work had grown so enormously that it was decided to no longer hold the meeting just preceding that of the American Medical Association, so the fifth annual meeting was held on May 23rd and 24th, 1911, at Philadelphia, in connection with the Philadelphia Milk Show. This was the largest meeting of its kind ever held in the United States, and the papers presented were of great scientific and practical value.

Thus, we see, this movement has become a National one with us, but international in its scope; other nations are aroused to the importance of this subject, and the profession the world over are lending their aid in securing pure, clean milk for our cities and patients. The time is not far distant when every city of 10,000 inhabitants will have "Certified Milk."

If by giving a few of the most important features in the development of this field of medical work, the essayist has aroused the interest of the profession to the importance of this great work and responsibility which has fallen to our lot to bear, and if the Fulton County Medical Society will respond to the appeal of dying infants and heartbroken mothers for pure, clean milk, and will place our city, the "First City in the South," in the front rank of progress by appointing a Medical Milk Commission to procure certified milk for our city and patients—then the object of this paper has been accomplished.

